# The Novel Long Noncoding RNA linc00467 Promotes Cell Survival but Is Down-Regulated by N-Myc

**DOI:** 10.1371/journal.pone.0088112

**Published:** 2014-02-19

**Authors:** Bernard Atmadibrata, Pei Y. Liu, Nicolas Sokolowski, Lihong Zhang, Matthew Wong, Andrew E. Tee, Glenn M. Marshall, Tao Liu

**Affiliations:** 1 Children's Cancer Institute Australia for Medical Research, Randwick, Sydney, Australia; 2 Department of Anatomy, Histology and Embryology, School of Basic Medical Sciences, Fudan University, Shanghai, China; 3 Kids Cancer Centre, Sydney Children's Hospital, Randwick, Australia; 4 School of Women's & Children's Health, UNSW Medicine, University of New South Wales, Randwick, Sydney, Australia; Harvard Medical School, United States of America

## Abstract

The worst subtype of neuroblastoma is caused by *MYCN* oncogene amplification and N-Myc oncoprotein over-expression. Long noncoding RNAs (lncRNAs) are emerging as critical regulators of gene expression and tumourigenesis. While Myc oncoproteins are well-known to exert tumourigenic effects by regulating the expression of protein-coding genes and microRNAs, little is known about which lncRNAs are Myc targets and whether the Myc target lncRNAs play a role in Myc-induced oncogenesis. Here we performed differential gene expression studies using lncRNA microarray in neuroblastoma cells after transfection with control or N-Myc-specific small interfering RNA (siRNA), and identified N-Myc target lncRNAs including the novel lncRNA linc00467, the expression and function of which were completely unknown. RT-PCR, chromatin immunoprecipitation and luciferase assays showed that N-Myc suppressed *linc00467* gene expression through direct binding to the *linc00467* gene promoter and reducing *linc00467* promoter activity. While N-Myc suppressed the expression of *RD3*, the protein-coding gene immediately down-stream of *linc00467* gene, through direct binding to the *RD3* gene promoter and reducing *RD3* promoter activity, linc00467 reduced RD3 mRNA expression. Moreover, Affymetrix microarray analysis revealed that one of genes significantly up-regulated by linc00467 siRNA was the tumour suppressor gene DKK1. Importantly, knocking-down linc00467 expression with siRNA in neuroblastoma cells reduced the number of viable cells and increased the percentage of apoptotic cells, and co-transfection with DKK1 siRNA blocked the effects. These findings therefore demonstrate that N-Myc-mediated suppression of *linc00467* gene transcription counterintuitively blocks N-Myc-mediated reduction in RD3 mRNA expression, and reduces neuroblastoma cell survival by inducing DKK1 expression.

## Introduction

Neuroblastoma is a solid extracranial paediatric cancer that arises from neural crest cells, and accounts for 15% of cancer-related death in children [1]. Amplification of *MYCN* oncogene and consequent N-Myc oncoprotein over-expression occur in approximately 40% of high risk neuroblastoma, and is clinically associated with cancer metastasis, resistance to therapies and poor patient outcome [1], [2].

Myc oncoproteins, including N-Myc and c-Myc, exert biological effects through modulating gene transcription. After Myc oncoproteins dimerize with Max, the Myc-MAX complex binds to Myc-responsive element E-boxes at target gene promoters, leading to transcriptional activation [3], [4]. On the other hand, Myc oncoproteins repress gene transcription by forming transcriptional repressor complexes with histone deacetylases at Sp1-binding sites of target gene promoters [5], [6], [7], [8]. Identifying N-Myc target genes and understanding the function of the N-Myc target genes are important in developing better anticancer therapies.

Long noncoding RNAs (lncRNAs) are transcripts longer than 200 nucleotides without a functional open reading frame, and can be divided into five different types: sense, antisense, bidirectional, intronic and intergenic (lincRNA) [9], [10]. lncRNAs are emerging as important regulators of gene transcription, tumour initiation and progression [9], [10]. For example, lincRNA-p21 is directly activated by p53 and functions as an inhibitor of the genes that interfere with apoptosis [11], the lincRNA CTBP1-AS promotes both hormone-dependent and castration-resistant prostate cancer growth [12], and the lincRNA MALAT1 and HOTAIR play critical roles in lung and breast cancer invasion and metastasis [13], [14].

Myc oncoproteins have been extensively shown to modulate the expression of microRNAs, and targeting the microRNAs is a promising approach for treating Myc-induced cancers (reviewed in [15]). However, little is known about which lincRNAs are Myc targets and whether the Myc target lincRNAs play a role in Myc-induced cancer. Here, we screened for lincRNA targets of N-Myc in neuroblastoma cells by noncoding RNA microarray, and identified linc00467 as an N-Myc target. While linc00467 had not been studied at all in the literature, we discovered that linc00467 suppressed the expression of its downstream protein-coding gene RD3, and induced neuroblastoma cell survival by reducing the expression of the tumour suppressor gene DKK1.

## Results

### N-Myc suppresses the expression of the long noncoding RNA linc00467 by direct binding to its gene promoter

Myc oncoproteins exert biological effects by modulating gene transcription. However, it is unknown whether N-Myc modulates the transcription of lncRNAs. We therefore performed differential gene expression analysis using NCode™ Human Non-coding RNA Microarray in BE(2)-C neuroblastoma cells 30 hours after transfection with control siRNA or N-Myc siRNA No. 1 (N-Myc siRNA-1). As shown in Table 1, the microarray gene expression study showed that 5 lncRNAs were down-regulated, and 1 lncRNA was up-regulated, by N-Myc siRNA-1 within 30 hours by more than 2 fold. One of the lncRNAs most significantly up-regulated by N-Myc siRNA-1 was *linc00467*, which was identified by Human Genome Organisation Gene Nomenclature Committee (HGNC) according to published DNA and cDNA sequencing data [16], [17], [18], [19], [20].

**Table 1:** Modulation of lncRNA expression by N-Myc siRNA-1 by more than 2 fold thirty hours after siRNA transfection, as identified by lncRNA microarray.

Probe name	Target ID	Probe Sequence	Fold change
h13721	AL122062	TACTTCTAAAAAAAGTATTTTGTATCTACTTTTGTAACTTCGTCAGAATAAAATATATTG; TTCGTCAGAATAAAATATATTG	0.33
h03377	AK002005	GTTATCCAGGAAACAATATATATACACTTGTGAACTGTTGTTTGTGATTTAAGCATATAT	0.36
h31926	uc002oww	TTGTAGATTGGTTGTGTTTACACAGTTGTATATATTGACACCCTTGAGTGTTATGACTTC	0.37
h33198	uc002vpg	AATATTCATTTCTGAAATACTTTAGTATGATAGATAAATTTGGTTAAGTTCTTGTTCATT	0.46
h17938	BC039246	ATGTACTAATAATTTTATCTGACTTCTGTTTATATCATTTGTACAATTACATGGTTGTAA	0.47
h26159	linc00467	GAAACAACCACATATGTCACCTTTCCAAGAGGGACTGAAACTGGGCTGACCCTTTTGATT	2.17

**Figure 1:**
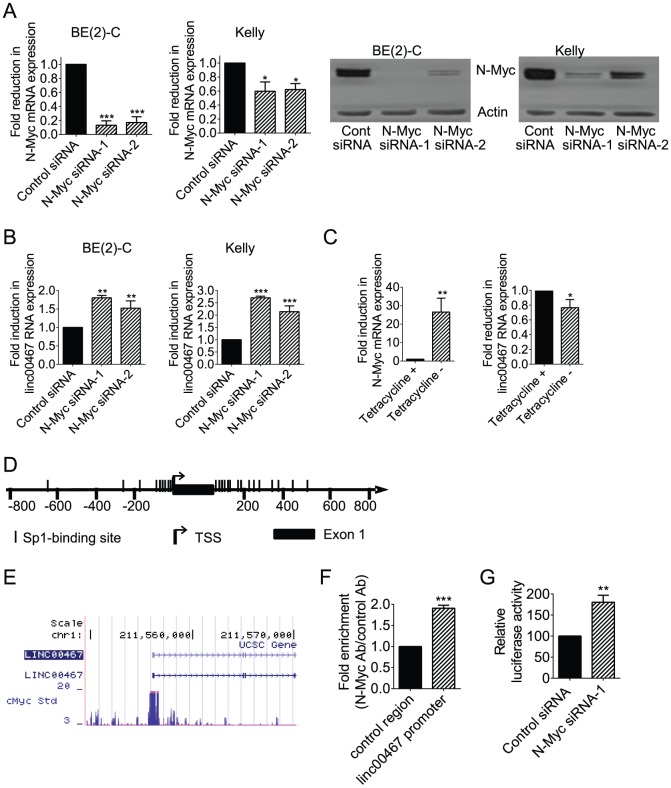
N-Myc represses *linc00467* gene expression by direct binding to the linc00467 gene promoter. (**A–B**). BE(2)-C and Kelly cells were transfected with scrambled control (Cont) siRNA, N-Myc siRNA-1 or N-Myc siRNA-2 for 48 hours, followed by RNA and protein extraction, real-time RT-PCR and immunoblot analyses of N-Myc mRNA, protein expression (**A**) or linc00467 RNA expression (**B**). (**C**) SHEP-21N cells were incubated with or without tetracycline for 48 hours, followed by RNA extraction and RT-PCR analysis of N-Myc and linc00467 RNA expression. (**D**) Schematic representation of the *linc00467* gene promoter. TSS represented transcription start site, and | represented Sp1-binding sites. (**E**) ChIP-Seq data from Dr. Michael Snyder's group at Yale University for the ENCODE/SYDH project generated from K562 cells. (**F**) ChIP assays were performed with a control or anti-N-Myc antibody (Ab) and primers targeting a negative control region or the *linc00467* gene core promoter region enriched in Sp1-binding sites in BE(2)-C cells. Fold enrichment was calculated by dividing PCR products from DNA samples immunoprecipitated with the anti-N-Myc Ab by PCR products from DNA samples immunoprecipitated with the control Ab, relative to input. Fold enrichment at the negative control region was artificially set as 1.0. (**G**) BE(2)-C cells were transfected with control siRNA or N-Myc siRNA-1, followed by co-transfection with Cypridina TK control construct plus empty vector or *linc00467* gene promoter pLightSwitch_Prom construct. Luciferase activities were measured with a LightSwitch Dual Assay System kit, and expressed as a percentage change relative to control siRNA transfected samples. Error bars represented standard error. *, ** and *** indicated *P*<0.05, 0.01 and 0.001 respectively.

To validate the microarray data, we performed siRNA transfections with control siRNA, N-Myc siRNA-1 or N-Myc siRNA-2 for 48 hours in two *MYCN* oncogene amplified human neuroblastoma cell lines, BE(2)-C and Kelly, followed by real-time RT-PCR study of linc00467. As shown in Figure 1A, transfection with N-Myc siRNA-1 or N-Myc siRNA-2 reduced the expression of both N-Myc mRNA and protein in the two neuroblastoma cell lines. Consistent with the microarray data, down-regulation of N-Myc expression resulted in increased linc00467 expression (Figure 1B). We next performed RT-PCR study of N-Myc and linc00467 in SHEP-21N neuroblastoma cells, which were stably transfected with a tetracycline withdrawal-inducible N-Myc expression construct [6], [7], 48 hours after incubation with or without tetracycline. As shown in Figure 1C, withdrawal of tetracycline induced N-Myc expression, and reduced linc00467 RNA expression.

We have shown previously that N-Myc represses gene transcription by recruiting histone deacetylases to Sp1-binding site-enriched regions of target gene promoters [5], [6], [7], [8]. To understand whether N-Myc could directly repress *linc00467* gene transcription, we firstly analysed transcription factor binding sites at the *linc00467* gene promoter with Gene-Regulation software (http://www.gene-regulation.com/pub/programs/alibaba2/index.html). Results showed that Sp1-binding sites were enriched at −176 bp to −14 bp upstream of *linc00467* gene transcription start site as well as +7 bp to +426 bp in intron 1 (Figure 1D). We then examined a c-Myc chromatin immunoprecipitation-sequencing (ChIP-Seq) dataset, which was generated by Dr. Michael Snyder's group at Yale University for the ENCODE/SYDH project (The Encyclopedia of DNA Elements/Stanford/Yale/USC/Harvard genome project). As shown in Figure 1E, the ChIP-seq data showed that c-Myc oncoprotein bound to the *linc00467* gene core promoter region matching the Sp1-binding site-enriched region in K562 leukemia cells. Consistently, our own ChIP assays showed that an anti-N-Myc antibody efficiently immunoprecipitated the region of the *linc00467* gene core promoter enriched in the Sp1-binding sites in BE(2)-C neuroblastoma cells (Figure 1F), in addition to the gene core promoter of ODC1 (Figure S1), a well-known Myc target gene. To further understand whether the binding of N-Myc to the *linc00467* gene promoter region repressed *linc00467* gene transcription, the Sp1-binding site-enriched region of the *linc00467* gene promoter was cloned into a pLightSwitch_Prom construct. Luciferase assays were performed in BE(2)-C cells after transfection with control siRNA or N-Myc siRNA-1, followed by transfection with a pLightSwitch_Prom construct expressing empty vector or the *linc00467* promoter region. Results showed that knocking-down N-Myc expression significantly up-regulated luciferase activity of the pLightSwitch_Prom construct expressing the *linc00467* promoter region (Figure 1G). Taken together, the data suggest that N-Myc represses *linc00467* gene transcription by direct binding to the Sp1-binding site-enriched region of the *linc00467* gene promoter and reducing *linc00467* promoter activity.

### Linc00467 reduces mRNA expression of the linc00467 neighbouring protein-coding gene RD3

**Figure 2:**
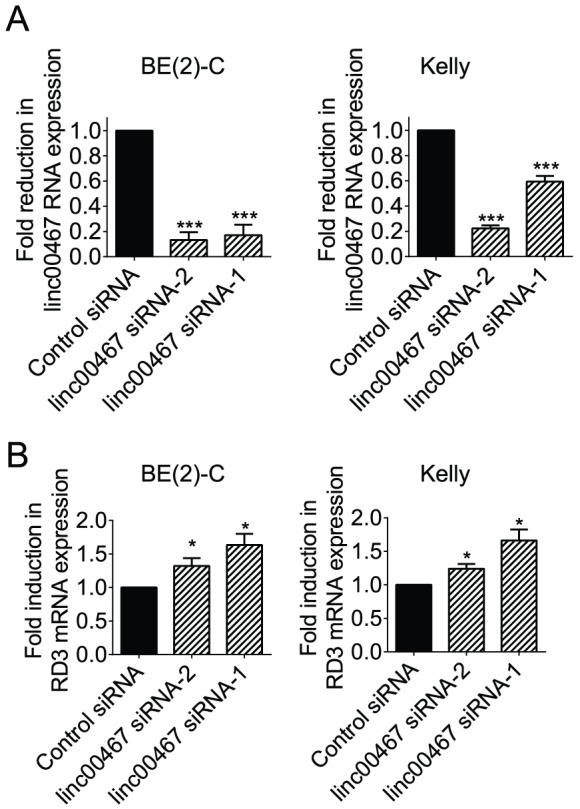
linc00467 reduces mRNA expression of its neighbouring protein-coding RD3. BE(2)-C and Kelly cells were transfected with scrambled control siRNA, linc00467 siRNA-1 or linc00467 siRNA-2 for 48 hours, followed by RNA extraction and and real-time RT-PCR analysis of the expression of linc00467 (**A**) or RD3 (**B**). Error bars represented standard error. * indicated *P*<0. 05, and *** indicated *P*<0.001.

lincRNAs exert biological functions partly through *in cis* regulation of mRNA expression of their neighbouring protein coding genes through various mechanisms [12], [21], [22], [23]. We therefore examined whether linc00467 regulated the expression of RD3, the gene immediately down-stream of linc00467. BE(2)-C and Kelly cells were transfected with control siRNA, linc00467 siRNA-1 or linc00467 siRNA-2 for 48 hours, followed by RT-PCR analysis of RD3 mRNA expression. As shown in Figure 2A, transfection with linc00467 siRNA-1 or linc00467 siRNA-2 reduced linc00467 RNA expression in the neuroblastoma cells. Importantly, knocking-down linc00467 expression up-regulated RD3 mRNA expression in both BE(2)-C and Kelly cells (Figure 2B). The data indicate that linc00467 reduces mRNA expression of its neighbouring protein-coding RD3.

### N-Myc represses *RD3* gene expression by direct binding to the *RD3* gene promoter

**Figure 3:**
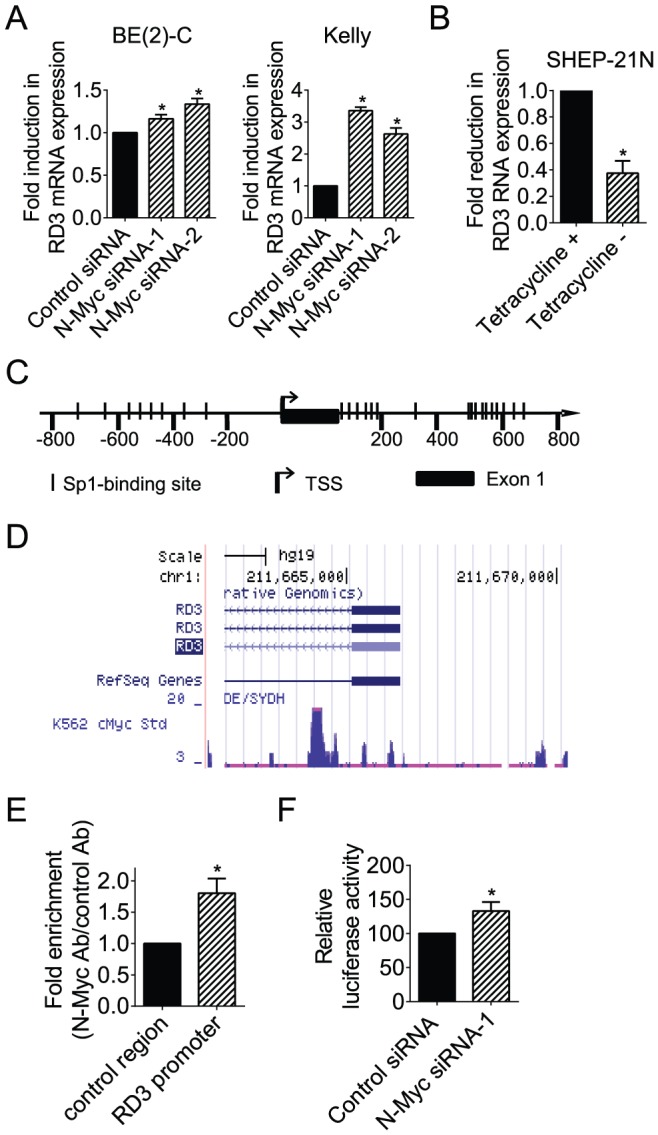
N-Myc represses *RD3* gene transcription by direct binding to the *RD3* gene promoter. (**A**) BE(2)-C and Kelly cells were transfected with scrambled control (Cont) siRNA, N-Myc siRNA-1 or N-Myc siRNA-2 for 48 hours, followed by RNA extraction and real-time RT-PCR analyses of RD3 mRNA expression. (**B**) SHEP-21N cells were incubated with or without tetracycline for 48 hours, followed by RNA extraction and RT-PCR analysis of RD3 RNA expression. (**C**) Schematic representation of the *RD3* gene promoter. TSS represented transcription start site, and | represented Sp1-binding sites. (**D**) ChIP-Seq data from Dr. Michael Snyder's group at Yale University for the ENCODE/SYDH project generated from K562 cells. (**E**) ChIP assays were performed with a control or anti-N-Myc antibody (Ab) and primers targeting a negative control region or the *RD3* gene core promoter region enriched in Sp1-binding sites in BE(2)-C cells. Fold enrichment was calculated by dividing PCR products from DNA samples immunoprecipitated with the anti-N-Myc Ab by PCR products from DNA samples immunoprecipitated with the control Ab, relative to input. Fold enrichment at the negative control region was artificially set as 1.0. (**F**) BE(2)-C cells were transfected with control siRNA or N-Myc siRNA-1, followed by co-transfection with Cypridina TK control construct plus empty vector or *RD3* gene promoter pLightSwitch_Prom construct. Luciferase activities were measured with a LightSwitch Dual Assay System kit, and expressed as a percentage change relative to control siRNA transfected samples. Error bars represented standard error. * indicated *P*<0.05.

p53 has been shown to indirectly repress the expression of a subset of genes by inducing the expression of lincRNA-p21 [11]. We therefore examined whether N-Myc increased RD3 mRNA expression indirectly by suppressing linc00467 expression. RT-PCR analysis of RD3 mRNA expression was performed in BE(2)-C and Kelly cells 48 hours after transfection with control siRNA, N-Myc siRNA-1 or N-Myc siRNA-2. Surprisingly, opposite to our expectation, knocking-down N-Myc expression with N-Myc siRNA-1 or N-Myc siRNA-2 up-regulated RD3 mRNA expression (Figure 3A). Consistent with this finding, withdrawal of tetracycline from SHEP-21N neuroblastoma cells, which induced N-Myc expression, reduced linc00467 RNA expression (Figure 3B). To understand whether N-Myc directly repressed *RD3* gene transcription, we firstly analysed transcription factor binding sites at the *RD3* gene promoter with Gene-Regulation software (http://www.gene-regulation.com/pub/programs/alibaba2/index.html.) Results showed that Sp1-binding sites were enriched at +475 bp to +731 bp of *RD3* gene intron 1, relative to intron 1 start site (Figure 3C). We then examined a c-Myc ChIP-Seq dataset, which was generated by Dr. Michael Snyder's group at Yale University for the ENCODE/SYDH project. As shown in Figure 3D, the ChIP-seq data showed that the c-Myc oncoprotein bound to the *RD3* gene intron 1 region matching the Sp1-binding site-enriched fragment in K562 leukemia cells. Consistently, our own ChIP assays with primers targeting *RD3* intron 1 showed that an anti-N-Myc antibody efficiently immunoprecipitated the *RD3* gene intron 1 region enriched in Sp1-binding sites in BE(2)-C neuroblastoma cells (Figure 3E). To further understand whether the binding of N-Myc to the *RD3* intron 1 region repressed *RD3* gene transcription, the Sp1-binding site-enriched *RD3* intron 1 region was cloned into a pLightSwitch_Prom construct. Luciferase assays were performed in BE(2)-C cells after transfection with control siRNA or N-Myc siRNA-1, followed by transfection with a pLightSwitch_Prom construct expressing empty vector or the *RD3* intron 1 region. Results showed that knocking-down N-Myc expression significantly up-regulated luciferase activity of the pLightSwitch_Prom construct expressing the RD3 intron 1 region (Figure 3F). To understand whether N-Myc and linc00467 co-operatively reduce RD3 expression, we transfected BE(2)-C cells with control siRNA, N-Myc siRNA, linc00467 siRNA, or combination of N-Myc siRNA and linc00467 siRNA. RT-PCR analysis showed that N-Myc siRNA and linc00467 siRNA did not have co-operative effect in modulating RD3 expression (Figure S2). Taken together, the data suggest that N-Myc represses *RD3* gene transcription by direct binding to the Sp1-binding site-enriched region of the *RD3* gene promoter and reducing *RD3* promoter activity.

### Knocking-down linc00467 expression reduces neuroblastoma cell survival

**Figure 4:**
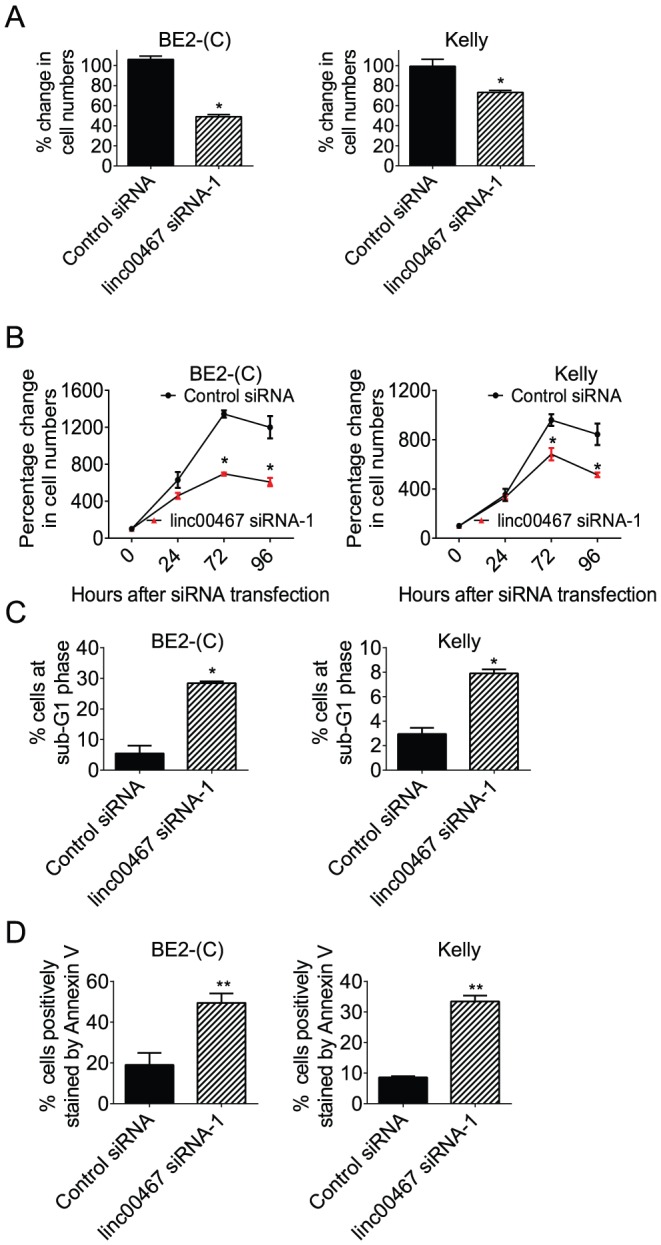
linc00467 enhances neuroblastoma cell survival. (**A**) BE(2)-C and Kelly cells were transfected with scrambled control siRNA or linc00467 siRNA-1 for 48 hours, followed by Alamar blue assays. The effect of linc00467 siRNA-1 was expressed as a percentage change in the number of viable cells after transfection with linc00467 siRNA-1, compared with control siRNA-transfected samples. (**B**) BE(2)-C and Kelly cells were transfected with scrambled control siRNA or linc00467 siRNA-1 for 0, 72 or 96 hours, followed by Alamar blue assays. The effects of time and siRNAs were expressed as percentages of the number of viable cells after transfection with control siRNA for 0 hour. (**C**) After transfection with control siRNA or linc00467 siRNA-1 for 72 hours, BE(2)-C and Kelly cells were stained with propodium iodide, and subjected to flow cytometry analyses of the cell cycle. The percentage of cells at sub-G1 phase was calculated. (**D**) After transfection with control siRNA or linc00467 siRNA-1 for 72 hours, BE(2)-C and Kelly cells were stained with FITC-conjugated Annexin V, and subjected to flow cytometry analyses. The percentage of cells positively stained by Annexin V was calculated. Error bars represented standard error. * indicated p<0.05, and ** p<0.01.

To understand whether repression of linc00467 expression by N-Myc contributed to an N-Myc-induced cancer phenotype, we transfected BE(2)-C and Kelly cells with control siRNA or linc00467 siRNA for 48 hours, followed by Alamar blue assays. As shown in Figure 4A, knocking-down linc00467 expression with siRNA reduced the number of viable BE(2)-C and Kelly cells. Alamar blue assays in BE(2)-C and Kelly cells 0, 24, 72 and 96 hours after transfection with control siRNA or linc00467 siRNA showed that linc00467 siRNA considerably reduced the number of viable cells 72 and 96 hours after siRNA transfection (Figure 4B). To examine whether the effect was due to cell death, we transfected BE(2)-C and Kelly cells with control siRNA or linc00467 siRNA, followed by staining with propidium iodide (PI) and cell cycle study with flow cytometry. We also transfected BE(2)-C and Kelly cells with control siRNA or linc00467 siRNA, followed by staining with the apoptosis marker fluorescein isothiocyanate (FITC)-conjugated Annexin V and analyses with flow cytometry. Data analyses showed that knocking-down linc00467 expression with siRNA increased the proportion of cells at sub-G1 phase of the cell cycle (Figure 4C) and the proportion of apoptotic cells (Figure 4D). Taken together, the data suggest that linc00467 promotes neuroblastoma cell survival.

### Reduction in DKK1 expression contributes to linc00467-mediated neuroblastoma cell survival

**Figure 5:**
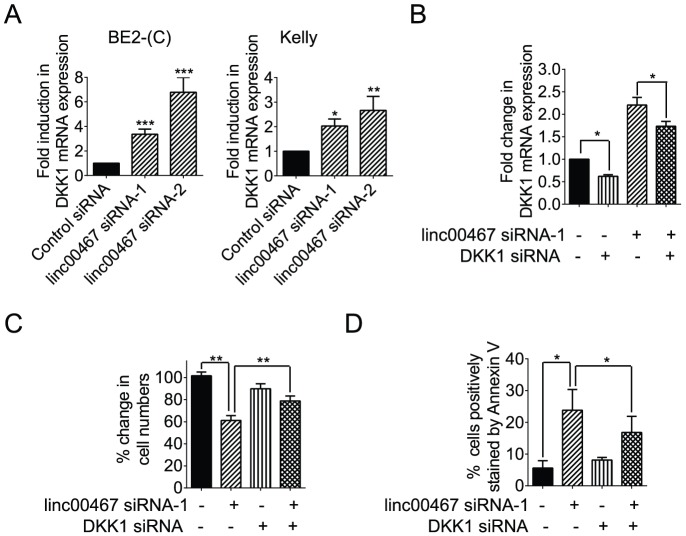
Reduction in DKK1 expression contributes to linc00467-mediated neuroblastoma cell survival. (**A**) BE(2)-C and Kelly cells were transfected with scrambled control siRNA, linc00467 siRNA-1 or linc00467 siRNA-2 for 48 hours, followed by RNA extraction and RT-PCR analysis of DKK1 gene expression. (**B**) BE(2)-C cells were transfected with scrambled control siRNA, linc00467 siRNA-1, DKK1 siRNA, or combination of linc00467 siRNA-1 and DKK1 siRNA for 48 hours, followed by RNA extraction and RT-PCR analysis of DKK1 gene expression. (**C**) BE(2)-C cells were transfected with scrambled control siRNA, linc00467 siRNA-1, DKK1 siRNA, or combination of linc00467 siRNA-1 and DKK1 siRNA for 72 hours, followed by Alamar blue assays. The effect of linc00467 siRNA-1 alone, DKK1 siRNA alone, or combination of linc00467 siRNA-1 and DKK1 siRNA was expressed as a percentage change, compared with control siRNA-transfected samples. (**D**) BE(2)-C cells were transfected with scrambled control siRNA, linc00467 siRNA-1, DKK1 siRNA, or combination of linc00467 siRNA-1 and DKK1 siRNA for 72 hours, followed by staining with FITC-conjugated Annexin V, and subjected to flow cytometry analyses. The percentage of cells positively stained by Annexin V was calculated. Error bars represented standard error. *, ** and *** indicated p<0.05, 0.01 and 0.001 respectively.

To understand the mechanism through which linc00467 promotes neuroblastoma cell survival, we performed differential gene expression study of linc00467 target genes in BE(2)-C cells 48 hours after transfection with control siRNA or linc00467 siRNA-1. As shown in Table S1, one of the genes significantly up-regulated by linc00467 siRNA-1 was DKK1, a Wnt antagonist tumour suppressor gene known to induce cancer cell apoptosis [24], [25]. RT-PCR analysis confirmed that transfection with linc00467 siRNA-1 or linc00467 siRNA-2 considerably up-regulated the expression of DKK1 in BE(2)-C and Kelly cells (Figure 5A).

To examine whether up-regulation of the tumour suppressor gene DKK1 contributed to linc00467 siRNA-mediated apoptosis, we transfected BE(2)-C cells with control siRNA, linc00467 siRNA-1, DKK1 siRNA, or combination of linc00467 siRNA-1 and DKK1 siRNA. RT-PCR analysis showed that DKK1 siRNA reduced DKK1 gene expression, and blocked linc00467 siRNA-mediated DKK1 gene up-regulation (Figure 5B). Alamar blue assays (Figure 5C) and flow cytometry analyses of Annexin V positively stained cells (Figure 5D) showed that linc00467 siRNA-1 reduced the number of viable cells and increased the proportion of cells positively stained by Annexin V, and that DKK1 siRNA blocked linc00467 siRNA-1-mediated reduction in the number of viable neuroblastoma cells and induction of Annexin V positively stained cells. Taken together, the data suggest that reduction in DKK1 expression contributes to linc00467-mediated neuroblastoma cell survival.

## Discussion

lncRNAs are emerging as critical regulators of gene transcription, tumour initiation, progression and metastasis [9], [10], [26]. Myc oncoproteins, including N-Myc and c-Myc, are well-known to exert biological effects by modulating the expression of protein-coding genes and microRNAs [3], [4], [15]. However, little is known about whether Myc modulates the expression of lncRNAs, and whether regulation of lncRNA expression by Myc plays a role in Myc oncogenesis. In this study, we have performed genome-wide differential expression study with lncRNA microarray in neuroblastoma cells 30 hours after knocking-down N-Myc gene expression. Data analysis reveals that knocking-down N-Myc gene expression for 30 hours alters the expression of 6 lncRNAs by more than 2 fold. One of the lncRNAs most significantly up-regulated by N-Myc siRNA is linc00467.

linc00467 was identified by Human Genome Organisation Gene Nomenclature Committee (HGNC) based on published DNA and cDNA sequencing data [16], [17], [18], [19], [20]. Till today, the biological function of linc00467 is completely unknown. We have found that the *linc00467* gene core promoter is enriched in Sp1-binding sites, and that c-Myc binds to the Sp1-binding site-enriched region of the *lin00467* gene core promoter in K562 leukemia cells according to a publically available ChIP-Seq dataset. Moreover, our own ChIP assays have confirmed that N-Myc indeed binds to the Sp1-binding site-enriched region of the *lin00467* gene core promoter, luciferase assays show that N-Myc siRNA enhances *linc00467* gene promoter activity, and RT-PCR data demonstrate that *linc00467* gene expression is reduced by N-Myc and up-regulated by N-Myc siRNAs. As N-Myc is well-known to repress gene transcription by direct binding to target gene promoter regions enriched in Sp1-binding sites [5],[6],[7],[8], our data suggest that N-Myc represses *linc00467* gene expression by direct binding to the *linc00467* gene promoter region enriched in Sp1-binidng sites and suppresses *linc00467* gene promoter activity.

lncRNAs exert biological effects through *in cis* and *in trans* regulation of RNA expression at both transcriptional and post-transcriptional levels. For examples, a number of lncRNAs have been shown to up- or down-regulate the expression of their neighboring protein-coding genes through modulating chromatin structure and gene transcription [14], [22], [27], [28]. The lncRNAs DLEU1 and DLEU2 at 13q14.3 are often deleted in multiple types of cancers, and DLEU1 and DLEU2 modulate nuclear factor B function by down-regulating the transcription of their neighboring protein-coding KPNA3 and the microRNAs miR-15 and miR-16 [23]. Additionally, the lncRNA MALAT1 controls cell cycle progression by regulating the expression of the oncogenic transcription factor B-MYB through altering the binding of splicing factors on B-MYB pre-mRNA and causing aberrant alternative splicing [29], and the PTEN pseudogene expressed noncoding RNA antisense RNA (PTENpg1 asRNA) regulates both *PTEN* gene transcription and PTEN mRNA stability [30]. In this study, we have confirmed that knocking-down linc00467 up-regulates the expression of its neighbouring protein-coding gene *RD3*, which encodes a retinal protein responsible for the retinal degeneration disorder Leber congenital amaurosis type 12 [31], [32]. Surprisingly, we have also confirmed that N-Myc suppresses *RD3* gene expression through direct binding to the *RD3* gene promoter region enriched in Sp1-binding sites and reducing *RD3* gene promoter activity. These data indicate that linc00467 reduces RD3 mRNA expression most likely through an *in cis* mechanism, and that N-Myc-mediated suppression of *linc00467* gene transcription counterintuitively blocks N-Myc-mediated reduction in RD3 mRNA expression. In addition, our differential gene expression study with Affymetrix microarray has identified DKK1 as one of the genes significantly up-regulated by linc00467 siRNA, suggesting that linc00467 is also likely to modulate gene expression through *in trans* mechanisms.

While the biological function of linc00467 is completely unknown in the literature, the Wnt antagonist DKK1 is well-known to induce cancer cell apoptosis and function as a tumour suppressor gene [24], [25]. This study reveals that knocking-down linc00467 gene expression reduces the number of viable neuroblastoma cells, increases the percentage of cells at sub-G1 phase of the cell cycle and induces apoptosis in neuroblastoma cells. Importantly, simultaneous knocking-down DKK1 expression blocks linc00467 siRNA-regulated neuroblastoma cell death. The data suggest that linc00467 may play a role in tumourigenesis through reducing DKK1 expression, leading to enhanced tumour cell viability, and that N-Myc-mediated suppression of *linc00467* gene transcription counterintuitively blocks N-Myc-mediated cell survival.

In summary, this study identifies lncRNAs as targets of N-Myc in neuroblastoma cells through genome-wide differential gene expression study, and demonstrates that N-Myc suppresses *linc00467* gene transcription through direct binding to the *linc00467* gene promoter. linc00467 reduces the expression of its neighbouring protein-coding gene *RD3*, while N-Myc suppresses *RD3* gene transcription through direct binding to the *RD3* gene promoter. Importantly, linc00467 enhances neuroblastoma cell survival through reducing DKK1 expression. These findings therefore demonstrate that N-Myc-mediated suppression of *linc00467* gene transcription counterintuitively blocks N-Myc-mediated reduction in RD3 mRNA expression, and reduces neuroblastoma cell survival by inducing DKK1 expression.

## Materials and Methods

### Cell culture

Neuroblastoma BE(2)-C cells were cultured in Dulbecco's modified Eagle's medium supplemented with 10% fetal calf serum [5], [6], [7], [8]. Kelly cells were grown in RPMI 1640 supplemented with 10% fetal calf serum and 1% L-glutamine.

### siRNA transfection

Cells were transfected with AllStars negative control siRNA, N-Myc siRNA, linc00467 siRNA or DKK1 siRNA (Qiagen) using Lipofectamine 2000 (Life Technologies) as previously described [5], [6], [7], [8].

### Real-time RT-PCR

Following siRNA transfections, RNA was extracted from cells using PureLink RNA Mini kit (Life Technologies) according to the manufacturer's instructions. RNA samples were then quantified using Nanodrop spectrophotometer and treated with DNAse 1 (Life Technologies) to remove remaining genomic DNA. Synthesis of cDNA from RNA samples was carried out using M-MLV Reverse Transcriptase (Invitrogen). Real-time RT PCR was performed in Applied Biosystems 7900 using SYBR green PCR Master Mix (Life Technologies) as previously described [5], [6], [7], [8].

### Immunoblot

For the analysis of protein expression by immunoblot, cells were lysed, protein extracted and separated by gel electrophoresis. After western transfer, membranes were probed with mouse anti-N-Myc antibody (1∶1000) (Santa Cruz Biotech), followed by horseradish peroxidase-conjugated anti-mouse (1∶10000) antiserum (Santa Cruz Biotech). Protein bands were visualized with SuperSignal (Pierce). The membranes were lastly re-probed with an anti-actin antibody (Sigma) as loading controls.

### lncRNA microarray

Neuroblastoma BE(2)-C cells were transfected with scrambled control siRNA or N-Myc siRNA-1. Thirty hours after siRNA transfection, RNA was extracted from the cells with RNeasy mini kit and treated with DNAse 1. Differential gene expression was examined with NCode™ Human Non-coding RNA Microarray (Invitrogene), according to the manufacturer's instructions. Results from the microarray hybridization were analysed with GeneSpring software (GeneSpring), and deposited at Gene Expression Omnibus website (accession number: GSE52984)

### mRNA microarray

Neuroblastoma BE(2)-C cells were transfected with scrambled control siRNA or linc00467 siRNA-1. Fourty-eight hours after siRNA transfection, RNA was extracted from the cells with RNeasy mini kit. Differential gene expression was examined with Affymetrix HuGene-2_0-st Arrays (Affymetrix), according to the manufacturer's instructions. Results from the microarray hybridization were analysed in R (http://www.r-project.org/) with bioconductor package (http://www.bioconductor.org/), and deposited at Gene Expression Omnibus website (accession number: GSE52985).

### Chromatin imunoprecipitation (ChIP) assays

ChIP assays were performed with an anti-N-Myc antibody (Merck Millipore) or a control antibody and PCR with primers targeting negative control region or Sp1-binding site-enriched region of the *linc00467* or *RD3* gene core promoter. Fold enrichment of the *linc00467* and *RD3* gene core promoter region by the anti-N-Myc antibody was calculated by dividing the PCR product from the *linc00467* or *RD3* gene core promoter region by the PCR product from the negative control region, relative to input.

### Luciferase assays

Sp1-binding site enriched *linc00467* gene promoter region (−248 bp upstream of *linc00467* gene transcription start site to +567 bp of intron 1) was custom-cloned into the pLightSwitch_Prom construct by SwitchGear Genomics. Sp1-binding site enriched intron 1 region of RD3 (0 bp to +1043 bp) was also custom-cloned into the pLightSwitch_Prom construct by SwitchGear Genomics. BE(2)-C neuroblastoma cells were transfected with control siRNA or N-Myc siRNA-1, followed by co-transfection with Cypridina TK control construct plus empty vector, *linc00467* or *RD3* gene promoter pLightSwitch_Prom construct. Luciferase activities were measured with a LightSwitch Dual Assay System kit (SwitchGear Genomics) according to the manufacturer's instructions, and expressed as percentage changes relative to control siRNA transfected samples.

### Alamar blue assays

Alamar blue assays were performed as previously described [33]. Briefly, cells were transfected with various siRNAs in 96 well plates. After siRNA transfection, cells were incubated with Alamar blue (Invitrogen), and the plates were read on a microplate reader at 570/595 nm. Results were calculated according to the optical density absorbance units and expressed as percentage change in viable cell number.

### Flow cytometry study

For the analysis of cells at sub-G1 phase, seventy-two hours after siRNA transfection, cells were harvested, fixed in 80% ethanol, washed and then stained with 50 µg/ml propidium iodide (Sigma) in solution containing 2 µg/ml RNase (Roche). Flow cytometric analysis of the cells was performed using FACS Calibur machine and FACS Diva software (BD Biosciences). The percentage of cells at sub-G1 phase of the cell cycle was analyzed.

For the analysis of apoptosis, seventy-two hours after siRNA transfection, cells were incubated with FITC-conjugated Annexin V (BD Biosciences), and then subjected to flow cytometric analysis of FITC-positive cells using FACS Calibur machine and FACS Diva software.

### Statistical analysis

Experiments were conducted 3 times in duplicates. Data were analysed on Prism 6 software (GraphPad) and presented as mean ± standard error. Differences were analyzed for significance using ANOVA among groups or two-sided unpaired t-test for two groups. A p value of less than 0.05 was considered statistically significant.

## Supporting Information

Figure S1 N-Myc directly binds to the ODC1 gene promoter. ChIP assays were performed with a control or anti-N-Myc antibody (Ab) and primers targeting a negative control region or the ODC1 gene core promoter region. Fold enrichment was calculated by dividing PCR products from DNA samples immunoprecipitated with the anti-N-Myc Ab by PCR products from DNA samples immunoprecipitated with the control Ab, relative to input. Fold enrichment at the negative control region was artificially set as 1.0. Error bars represented standard error. ** indicated *P*<0. 01.
(PDF)

Figure S2 N-Myc and linc00467 do not have a co-operative effect on RD3 expression in neuroblastoma cells. BE(2)-C cells were transfected with scrambled control siRNA, N-Myc siRNA-1, N-Myc siRNA-2, linc00467 siRNA-1, linc00467 siRNA-2, combination of N-Myc siRNA-1 and linc00467 siRNA-1, or combination of N-Myc siRNA-2 and linc00467 siRNA-2 for 48 hours, followed by RT-PCR analysis of RD3 expression. Error bars represented standard error. * indicated *P*<0. 01, compared with control siRNA-transfected samples.
(PDF)

Table S1 Modulation of target gene expression by linc00467 siRNA-1 by more than 1.8 fold, as identified by Affymetrix microarray, in BE(2)-C cells 48 hours after transfection with control siRNA or linc00467 siRNA-1. The cut-off was set at 1.80 fold, as linc00467 siRNA-1 reduced the expression of linc00467 by 1.841 fold.
(DOCX)
