# Supplementary figures and images for: The Novel Long Noncoding RNA linc00467 Promotes Cell Survival but Is Down-Regulated by N-Myc
Source: PLoS One. 2014 Feb 19;9(2):e88112. doi: 10.1371/journal.pone.0088112 (PMC3929584; doi:10.1371/journal.pone.0088112)

## Supporting Information Figure S1

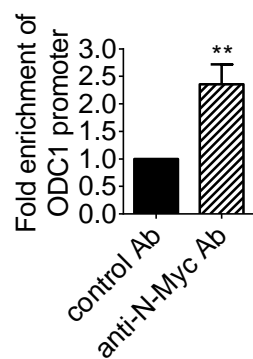

Supplement: Figure S1 — N-Myc directly binds to the ODC1 gene promoter. ChIP assays were performed with a control or anti-N-Myc antibody (Ab) and primers targeting a negative control region or the ODC1 gene core promoter region. Fold enrichment was calculated by dividing PCR products from DNA samples immunoprecipitated with the anti-N-Myc Ab by PCR products from DNA samples immunoprecipitated with the control Ab, relative to input. Fold enrichment at the negative control region was artificially set as 1.0. Error bars represented standard error. ** indicated P<0. 01. (PDF) [file pone.0088112.s001.pdf]

Supporting Information Figure S2

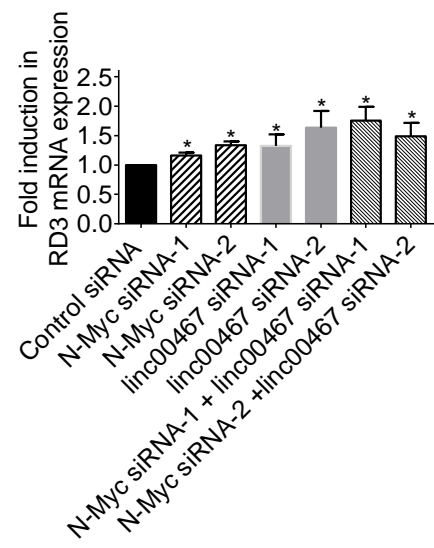

Supplement: Figure S2 — N-Myc and linc00467 do not have a co-operative effect on RD3 expression in neuroblastoma cells. BE(2)-C cells were transfected with scrambled control siRNA, N-Myc siRNA-1, N-Myc siRNA-2, linc00467 siRNA-1, linc00467 siRNA-2, combination of N-Myc siRNA-1 and linc00467 siRNA-1, or combination of N-Myc siRNA-2 and linc00467 siRNA-2 for 48 hours, followed by RT-PCR analysis of RD3 expression. Error bars represented standard error. * indicated P<0. 01, compared with control siRNA-transfected samples. (PDF) [file pone.0088112.s002.pdf]
