# Supplementary material for: The Novel Long Noncoding RNA linc00467 Promotes Cell Survival but Is Down-Regulated by N-Myc
Source: PLoS One. 2014 Feb 19;9(2):e88112. doi: 10.1371/journal.pone.0088112 (PMC3929584; doi:10.1371/journal.pone.0088112)
Supplement: Table S1 — Modulation of target gene expression by linc00467 siRNA-1 by more than 1.8 fold, as identified by Affymetrix microarray, in BE(2)-C cells 48 hours after transfection with control siRNA or linc00467 siRNA-1. The cut-off was set at 1.80 fold, as linc00467 siRNA-1 reduced the expression of linc00467 by 1.841 fold. (DOCX) [file pone.0088112.s003.docx]

**Supporting Information Table S1**.

| Probe ID | Fold change | Gene Symbol |
| --- | --- | --- |
| 16826273 | -4.739 | ITFG1 |
| 17100390 | -3.233 | TMEM203 |
| 16819207 | -2.869 | MT2A |
| 16819244 | -2.844 | MT1CP |
| 16805870 | -2.568 | NBEAP1 |
| 16768854 | -2.497 | ELK3 |
| 16774724 | -2.470 | CYSLTR2 |
| 17063066 | -2.469 | EEF1G |
| 16771303 | -2.427 | TRIAP1 |
| 17111874 | -2.407 | SNX12 |
| 17118690 | -2.399 | SNHG12 |
| 16995925 | -2.384 | C5orf28 |
| 16760905 | -2.317 | FAM86FP |
| 16966186 | -2.317 | N4BP2 |
| 16986244 | -2.290 | FAM169A |
| 16800058 | -2.285 | CAPN3 |
| 16860418 | -2.276 | CCNE1 |
| 16819252 | -2.248 | MT1F |
| 16826738 | -2.226 | MT1G |
| 16924878 | -2.206 | TIAM1 |
| 16904667 | -2.205 | SCN9A |
| 16713057 | -2.187 | ARHGAP12 |
| 16849923 | -2.170 | FAM195B |
| 16815407 | -2.169 | OR1F1 |
| 17005473 | -2.167 | SCGN |
| 16718922 | -2.164 | PRDX3 |
| 17063507 | -2.159 | JHDM1D |
| 16666965 | -2.151 | LRRC8B |
| 16769440 | -2.146 | PGAM1 |
| 16767851 | -2.130 | E2F7 |
| 16736226 | -2.112 | PIK3C2A |
| 16764564 | -2.110 | RIMA1 |
| 16763467 | -2.097 | PLEKHA8P1 |
| 16928967 | -2.093 | MTFP1 |
| 16816178 | -2.081 | MPV17L |
| 17016496 | -2.074 | HIST1H2AK |
| 17112560 | -2.069 | NAP1L3 |
| 16852445 | -2.069 | C18orf54 |
| 16996545 | -2.068 | DEPDC1B |
| 16758242 | -2.063 | BCL7A |
| 16706933 | -2.062 | FAM35A |
| 16827366 | -2.059 | ATP6V0D1 |
| 17117583 | -2.050 | EIF4B |
| 16809326 | -2.036 | LYSMD2 |
| 16878335 | -2.026 | ZNF512 |
| 16846121 | -2.046 | MRPL10 |
| 16820398 | -2.020 | SLC7A6 |
| 17067410 | -2.013 | FZD3 |
| 17021217 | -2.005 | ME1 |
| 16857608 | -2.003 | ZNF358 |
| 16724087 | -2.000 | TSPAN18 |
| 16988921 | -1.999 | LYRM7 |
| 16743432 | -1.989 | SESN3 |
| 16847117 | -1.978 | MTMR4 |
| 16890602 | -1.978 | PKI55 |
| 17057464 | -1.977 | SEPT7P2 |
| 17080516 | -1.974 | ENPP2 |
| 17111594 | -1.974 | SPIN4 |
| 16916312 | -1.968 | TBC1D20 |
| 16699138 | -1.968 | ANGEL2 |
| 17110616 | -1.968 | TBC1D25 |
| 16826539 | -1.966 | AKTIP |
| 16781431 | -1.951 | OR4N2 |
| 16842637 | -1.951 | PIGS |
| 16917602 | -1.948 | RBBP9 |
| 16713897 | -1.940 | PTPN20B |
| 16793176 | -1.939 | GCH1 |
| 16762996 | -1.928 | H3F3C |
| 16803103 | -1.927 | MPI |
| 17044905 | -1.917 | ADCYAP1R1 |
| 16819469 | -1.915 | ARL2BP |
| 16769649 | -1.911 | PRDM4 |
| 16681511 | -1.910 | LZIC |
| 16795128 | -1.908 | TMED8 |
| 16856887 | -1.906 | GNA11 |
| 17038267 | -1.902 | MSH5 |
| 17049904 | -1.898 | LRRC17 |
| 16917004 | -1.897 | GPCPD1 |
| 16932753 | -1.887 | LOC100132705 |
| 16957824 | -1.880 | COX17 |
| 16936137 | -1.877 | CERK |
| 16763280 | -1.876 | ZCRB1 |
| 16745583 | -1.876 | SCN3B |
| 17086640 | -1.870 | SECISBP2 |
| 16924862 | -1.865 | KRTAP21-2 |
| 16779956 | -1.863 | SLAIN1 |
| 17012342 | -1.862 | HINT3 |
| 17098491 | -1.861 | RPL12 |
| 16845126 | -1.853 | STAT3 |
| 16828462 | -1.850 | TMEM170A |
| 16762921 | -1.847 | FAM60A |
| 17073994 | -1.842 | C8orf33 |
| 16677153 | -1.841 | LINC00467 |
| 16771894 | -1.841 | MPHOSPH9 |
| 17016692 | -1.839 | UBD |
| 16667516 | -1.838 | LPPR4 |
| 17097340 | -1.837 | INIP |
| 17101447 | -1.835 | HCCS |
| 16911040 | -1.834 | CDC25B |
| 16835386 | -1.830 | SNX11 |
| 17067605 | -1.829 | LOC100507341 |
| 17116338 | -1.826 | CDY2A |
| 17016205 | -1.819 | C6orf62 |
| 16848739 | -1.818 | HN1 |
| 16970998 | -1.807 | USP38 |
| 16795582 | -1.806 | PTPN21 |
| 16921948 | -1.803 | USP16 |
| 16878731 | -1.802 | EHD3 |
|  |  |  |
| 17072979 | 1.801 | KHDRBS3 |
| 17118035 | 1.808 | CRMP1 |
| 16856216 | 1.814 | HCN2 |
| 16802479 | 1.815 | GLCE |
| 16679546 | 1.830 | DESI2 |
| 17016296 | 1.833 | HIST1H2AA |
| 16834111 | 1.839 | TMEM99 |
| 16699106 | 1.845 | BATF3 |
| 16705011 | 1.846 | DKK1 |
| 16662475 | 1.862 | EIF2C3 |
| 17093949 | 1.863 | RNF38 |
| 16722720 | 1.871 | NAV2 |
| 17088100 | 1.880 | UGCG |
| 16945543 | 1.884 | COL6A6 |
| 17104908 | 1.885 | CHIC1 |
| 17098594 | 1.889 | ENG |
| 16994743 | 1.891 | CDH12 |
| 16681891 | 1.905 | PRAMEF3 |
| 16864829 | 1.905 | ZNF610 |
| 16865900 | 1.912 | ZNF583 |
| 17053892 | 1.931 | INSIG1 |
| 16869684 | 1.967 | EMR2 |
| 16876324 | 1.982 | LOC100289079 |
| 17088148 | 1.983 | SNX30 |
| 16709245 | 1.994 | ADRA2A |
| 16924920 | 2.001 | KRTAP19-8 |
| 16666392 | 2.015 | FAM73A |
| 17061099 | 2.060 | RASA4B |
| 16764220 | 2.084 | RHEBL1 |
| 16667760 | 2.126 | S1PR1 |
| 17096205 | 2.134 | ZNF367 |
| 16731068 | 2.148 | LAYN |
| 16858451 | 2.158 | LPPR2 |
| 16998551 | 2.180 | SLCO4C1 |
| 17087517 | 2.190 | NR4A3 |
| 16876286 | 2.198 | KIR2DS2 |
| 16670479 | 2.217 | C1orf51 |
| 16996605 | 2.246 | C5orf43 |
| 16803533 | 2.270 | AGPHD1 |
| 16854540 | 2.286 | B4GALT6 |
| 16877133 | 2.366 | PQLC3 |
| 17023799 | 2.392 | SLC2A12 |
| 16784256 | 2.403 | STYX |
| 16874339 | 2.245 | RRAS |
| 16848079 | 2.585 | WIPI1 |
| 17065851 | 2.614 | FAM66D |
| 17125798 | 2.689 | LOC100132147 |
| 16768406 | 2.945 | LUM |
